# Psychometric properties and measurement invariance of Short-Form Life Attitude Inventory for hospital staff

**DOI:** 10.1186/s12909-022-03450-3

**Published:** 2022-05-30

**Authors:** Kang-Ju Chou, Ying-Yao Cheng, Hua-Chang Fang, Fu-Zong Wu, Pei-Chin Lin, Chun-Teng Tsai

**Affiliations:** 1 Institute of Education, National Sun Yat-sen University, Kaohsiung, 813 Taiwan; 2 School of Medicine, National Yang Ming Chiao Tung University, Taipei, Taiwan 112; 3 Department of Internal Medicine, Kaohsiung Veterans General Hospital, Kaohsiung, Taiwan 813; 4 Division of Medical Education, Kaohsiung Veterans General Hospital, Kaohsiung, Taiwan 813

**Keywords:** Psychometric properties, Life Attitude Inventory, Confirmatory factor analysis, Invariance

## Abstract

**Background:**

The life attitude of health care workers can deeply influence the quality of care. Examining the performance of the Short-Form Life Attitude Inventory (SF-LAI), this study analyzes the factorial structure, reliability, and invariance of the revised SF-LAI across genders and professions among the staff of a teaching medical center.

**Methods:**

The SF-LAI was developed for university students in Taiwan. From January to February 2019, we administered a cross-sectional survey of life attitudes by distributing the SF-LAI to all staff members of a medical center in Taiwan. The construct validity was evaluated using a confirmatory factor analysis (CFA). Model fit was assessed in terms of the comparative fit index (CFI), Tucker–Lewis index (TFI), standardized root mean square residual (SRMR), and root mean square of error of approximation (RMSEA). Internal consistency was calculated using Cronbach’s alpha and McDonald’s omega. We also performed the CFA invariance analysis for the SF-LAI-R across genders and professions (physician, nurse and other hospital staff).

**Results:**

Of 884 (24.62%) responses, 835 were valid. The participants had a mean age of 47.8 years, and 20.12% were male. In a comparison of multiple CFAs, a second-order model with six factors outperformed other models. The goodness of fit indices revealed the CFI was 0.955, TFI was 0.952, RMSEA was 0.071, and SRMR was 0.038. The Cronbach’s alphas, McDonald’s omega coefficients for internal consistency were all greater than 0.8. The first and second-order model had metric and scalar invariance across genders and professions.

**Conclusions:**

As health care demands evolve, humanities are becoming more important in medical education. Life attitude of hospital care worker is a crucial indicator of whether one embodies the ideals of a humanistic education. The revised SF-LAI has acceptable structural validity, internal consistency, and invariance across genders and professions among staff members of a teaching medical center.

## Background

According to the data released by the United Nations Population Division, the percentage of the global population aged ≥ 65 will almost double over the next 30 years, increasing from 9.1% to 15.9% of the global population [1]. This implies that the average patient will have more comorbidities in the future. In addition to having more than one systemic disease, the patient may also experience psychological, spiritual, and social difficulties [2, 3].

The provision of so-called whole person care (WPC) is crucial to meet the needs of these patients because it attends to the patient’s full spectrum of needs, including medical, behavioral, and socioeconomic [4–6]. WPC yields improved clinical outcomes, increased care quality, reduced cost of care, and greater consumer satisfaction [7–9].

WPC has been an objective of healthcare reform and medical education in the recent 20 years [10–12], but it is difficult to put into practice despite having theoretical principles that are easy to understand [13]. Transformative education was proposed to propagate WPC [14]. Because every decision is based on a person’s beliefs, attitudes, and values, whether held consciously or unconsciously [15], convincing healthcare workers (HCWs) to adopt WPC is crucial to encouraging them to reflect on the meaning of life to shape a positive attitude and empathy for life [16, 17]. Several studies have demonstrated that the attitudes, beliefs, values, and norms of HCWs play an essential role in the health care experiences and treatment outcomes of patients [18, 19]. In addition to affecting their interaction with patients, the attitudes and beliefs of HCWs can also influence their motivation to alter their own practices and behaviors at work [20–27]. HCWs in teaching hospitals often observe birth, senility, sickness, and death and accompany people through momentous life events. The effect of these experiences on HCW attitudes has not been explored, and no reliable instrument exists to measure it.

### Measuring attitude toward life: Short-Form Life Attitude Inventory

Life attitude refers to a person’s perceptions of the purpose of one’s life, control over one’s life, presence of an existential vacuum, acceptance of death, will to find meaning in life, and the pursuit of one’s goals [28]. The more positive one's life attitude is, the more one can accept frustrations and experience being loved and cared for [29].

In 1981, Reker and Peacock developed the self-reported Life Attitude Profile (LAP) to assess meaning in life (MiL) from logotherapeutic assumptions [28]. Originally, it contained 7 factors and 56 items. In 1992, Reker proposed the revised version, Life Attitude Profile-Revised, LAP-R [30], which contains 48 items for assessing 6 dimensions of MiL: purpose (having life goals and a sense of direction from the past, in the present, and toward the future), coherence (having a sense of order, a reason for existence, and a clear sense of personal identity), choice or responsibility (perception of freedom to make all life choices for oneself and take responsibility), acceptance of death (fearlessness of death and acceptance as a natural aspect of life), existential vacuum (lack of sense and orientation in life), and goal seeking (desire to search for new and diverse experiences).

Several studies have analyzed the psychometric properties of the LAP-R when applied to individuals from various countries and populations, including adolescents, college students, and patients with cancer [31–34]. These studies have reported varying results, and the proposed factorial structures have ranged from three to six. Some LAP-R scales have exhibited satisfactory internal consistency, and others did not. The results have suggested that implementing LAP necessitates a consideration of cross-cultural elements and the particularities of a given population.

Some life attitude scales have been formulated for the Taiwanese population (Leung M, Steinfort T, Vroon EJ: Life attitudes scale: Development and validation of a measurement of the construct of tragic optimism, Unpublished) [35–37]; however, most are person-centered and focus on psychotherapy. In 2010, Hsieh and Pan developed a Life Attitude Inventory (LAI) in traditional Chinese to assess university students’ attitude toward life in Taiwan; the LAI is based on the concepts of life formulated by Jean-Paul Sartre, Viktor Frankl, Rollo May, and Carl Rogers [38]. The LAI comprises 70 items in 6 dimensions: ideals of life (having meaningful life goals and worthy of effort to fulfill), autonomy (perception of freedom to make life choices for oneself and take responsibility), love and care (perception of others’ existence and being altruistic), feeling of existence (being sure of the meaning and value of their existence), attitude toward death (expectations, attitudes, and behaviors toward death), and life experience (attitude and reactions toward life’s setbacks and sufferings). The six dimensions can be categorized into three relationships: with oneself, with others, and with their situation. The ideals of life, autonomy, and feeling of existence were included in the relationships with self and love and care in the relationships with others, attitude toward death, and life experience in one’s relationship with their situation. Because the work of HCWs is almost entirely about others and their life situations, the LAI is more suited to measuring HCWs’ attitudes toward life than other scales are. In 2015, Hsieh and Pan proposed the simplified version, the Short-Form LAI (SF-LAI) [39]. For each dimension, they selected four items with improved reliability. The SF-LAI had a Cronbach’s alpha of 0.93 in a psychometric analysis. The reliability estimates for all factors ranged from 0.68–0.80. A confirmatory factor analysis (CFA) indicated that the six-factor model had a good fit, at χ^2^ (237) = 1078.58, χ^2^/df = 4.55, GFI = 0.93, CFI = 0.93, and RMSEA = 0.053 [37]. Although this Taiwanese version of LAI had cross-cultural validity and suited the requirements for measuring the life attitudes of HCWs, the structural validity and internal consistency among HCWs has yet to be determined.

We conducted this study to (1) analyze the applicability of the SF-LAI to the HCWs of a teaching medical center, (2) explore the validity and internal reliability of the revised version of the SF-LAI, and (3) examine the extent of measurement invariance across genders and professions.

## Methods

### Study design and ethics

This study was carried out at a 1400-bed teaching medical center in Taiwan. After obtaining permission from the authors to use the SF-LAI, we distributed an explanatory statement to all hospital staff about the study as well as guarantees of the anonymity and confidentiality of all information submitted through the institutional email system once per month from January to February 2019. Staff who consented to respond to the inventory could access the online version of the SF-LAI through a link in the email and could self-report their answers to the questionnaire. Data on sociodemographic variables, including gender, age group, type of identity, and job category, were also collected. Each item in the inventory required an answer, but participants could withdraw from answering the questionnaire at any time without having their information recorded. Data collection lasted for 3 months from January to March 2019, and only data from completed and submitted questionnaires were analyzed.

This study was approved by the ethics committee of Kaohsiung Veterans General Hospital, and the procedures were conducted in accordance with the Declaration of Helsinki.

### Instrument

The SF-LAI, a 24-question instrument that evaluates 6 dimensions of life attitude using a 7-point Likert scale (1: *strongly disagree*; 7: *strongly agree*), was used in the present study.

### Data quality and descriptive statistics

To assess item variability, we calculated the mean, standard deviations (SDs), central tendency, and skewness for each item. A skewness and kurtosis within a range of ± 2 indicated normality [40]. The criteria for the good–poor analysis (differences between the highest and lowest scoring groups of items) was *P* < 0.05, and the item–total analysis (the correlation coefficient between the item and the total score) was ≥ 0.5 [41].

### Goodness of fit test

Although diagonally weighted least squares (DWLS) estimator seems to be ideal for handling ordinal data [42]. In our study, the number of categories(ordinal) was large (> 5), no missing data was identified [43], and maximum likelihood (ML) has been proposed to have acceptable relative bias and relative standard error bias in CFA of mixed format data [44]. Thus, Amos software (version 27.0) with ML was used to conduct CFA) to verify the construct validity of the SF-LAI. In addition to relative (normed) chi square statistics (**χ**^**2**^/df) as a measure of fit, values < 5 indicated an acceptable fit and values < 3 indicated a good model fit [45]. Four conventional indices of goodness of fit were calculated: the comparative fit index (CFI) and the Tucker–Lewis index (TLI), with values ≥ 0.90 indicating acceptable fit and values ≥ 0.95 indicating good model fit [46]; the root mean square error of approximation (RMSEA), and the standardized root mean square residual (SRMR), with values ≤ 0.08 indicating acceptable and values ≤ 0.05 indicating good model fit [47, 48].

The original six-factor structure model was tested using a CFA. If modifications were used, they were minimized and based on statistical and theoretical concerns; problematic items were eliminated, according to Anderson and Gerbing’s recommendation [49]. A second-order factor analysis was conducted to examine any latent variables in the first stage and more general concepts in the second stage [50]. The target coefficient (T), which was the ratio of the chi square of the first-order model to the chis-square of the higher order (more restrictive) model, was used to evaluate whether the first- or second-order model is preferable [51], where *T* = 1 and *T* ≥ 0.75 indicated perfect and reasonable fit, respectively [52].

### Construct validity of the assessment tool

Construct validity refers to an extent to which the measurement score reflects latent construct to be measured [53]. According to Fornell and Larcker construct validity of CFA includes convergent validity test and discriminant validity test [54]. Convergent validity refers to the degree to which similar constructs are measured with different variables. It is based on the correlation between responses of different variables in measuring the same construct [55]. We assessed convergent validity in terms of standardized factor loading, and the average variance extracted (AVE). Factor loading ≥ 0.50 [56] and AVE ≥ 0.50 [57] are recommended as acceptable convergent validity.

Discriminant validity was assessed using the following two strategies [54]. One was Chi-square difference test. The model was constructed for each of the fifteen possible paired correlations between the latent variables. Then it was analyzed with (a) the correlation between the latent variables fixed at a value of 1 and (b) the correlation between the latent variables free to assume any value. The difference in chi-square values for the fixed and free solutions are believed to indicate whether a unidimensional model is sufficient to account for the intercorrelations among the observed variables in each pair [58]. The other was using bootstrapping approaches with 1000 samples to test the standard error of correlation coefficients between the six latent variables. A 95% confidence interval (CI) was calculated for the upper and lower bounds of the correlation coefficients (ϕ ± 2σ_e_). If the 95% CI does not contain 1.0, the pair of latent variables is considered discriminative [59].

### Reliability

Because our study was cross-sectional online and anonymous, it is difficult to do test–retest. To assess reliability, we evaluated internal consistency reliability with Cronbach’s alpha (CA), and McDonald’s omega total (ω_*t*_) and hierarchical (ω_*h*_) coefficients. CA was to measure how well each individual item in a subscale correlated with the sum of the remaining items [60], McDonald’s ω_*t*_, like CA, but don’t assume essential tau-equivalence, which is based on factor analysis, was used to separate the shared variance between the items from the single variance [61]. Both CA and ω_*t*_ are based on the assumption of unidimensionality. Hierarchical omega extends the utility for estimating the internal consistency reliability of scores on a multidimensional scale [61], include higher-order scales. The threshold of statistical measure in reliability validity is CA and McDonald’s ω_*t*_ ≥ 0.70 [57], ω_*h*_ ≥ 0.65 [62].

### Measurement invariance

To assess validity and applicability across various subpopulations, we conducted multigroup CFA by dividing the sample by gender and medical profession (physician, nurse, and other hospital staff) and performing separate subgroup CFAs. We compared five models [63]: model 1, configural invariance, included no cross-groups constraints, model 2 was used to test for metric invariance of the first-order factors, model 3 was used to test for metric invariance of the second-order factors, and models 4 and 5 were used to test for full scalar invariance of the first- and second-order factors in the model. The model was considered to be invariant across the groups if the difference in CFI and TLI between the unconstrained model and the weight-constrained model was less than 0.01 and 0.05, respectively [64, 65].

All analyses were performed with IBM SPSS ver. 27.0 and Amos 27.0 (IBM Corp. Armonk, NY, USA).

## Results

### Demographic data

The inventory was completed by 884 hospital staff members, and 835 valid responses were collected, for a 24.8% response rate. The participants comprised those from all professions in the hospital. Men, physicians, and new staff (who worked less than 5 years) had a significantly lower response rate. The demographic data are presented in Table 1.

**Table 1:** Demographic characteristics of study participants (*n* = 835)

Variable		Total (*n* = 835)		Response rate % (total n.)	*P*-value for response rate
Gender		N	%		< 0.001
	Male	168	20.12	18.32 (917)	
	Female	667	79.88	24.94 (2674)	
Age (y/o)					0.593
	< 30	209	25.03	21.75 (961)	
	30–39	232	27.78	21.52 (1078)	
	40–49	199	23.83	26.60 (748)	
	≥ 50	195	23.34	24.25 (804)	
Medical profession					0.032
	Physician	99	11.86	13.51^*^(733)	
	Nurse	436	52.22	26.76 (1629)	
	Other medical profession	113	13.53	28.25 (400)	
	administrative	187	22.40	22.56 (829)	
Working years (y)					0.033
	< 5	247	29.58	20.33*(1215)	
	5–9	201	24.07	24.07 (835)	
	10–19	150	17.96	24.15 (621)	
	≥ 20	237	28.38	25.76 (920)	

^a^*P* values are for chi squared tests for proportions and t-tests for means

^*^P < .05 compares with the other groups

### Internal structure

Table 2 displays the means, SDs, skewness, kurtosis, and the item–total correlation of items. The absolute values of skewness and kurtosis were less than 1. The results of the good–poor and item–total analyses all met the criteria. Therefore, no item was removed.

**Table 2:** Descriptive statistics of SFLAI-R

No	Item	Mean	SD	Skewness (SE)	Kurtosis (SE)	Item-Total correlation^a^
1	I believe that I have a dream to fulfill 我相信在這個世界上, 有一個等待我去實現 的夢想。	**5.15**	**1.18**	**-0.46(0.09)**	**0.19(0.17)**	**0.628**
2	I know what kind of life I want to lead 我知道什麼是我想要的生活。	**5.22**	**1.07**	**-0.51(0.09)**	**0.17(0.17)**	**0.663**
3	I am enthusiastic about pursuing my life goal 我勇於追求我想要的生活目標。	**5.12**	**1.06**	**-0.34(0.09)**	**-0.01(0.17)**	**0.719**
4	Living according to my values makes my life meaningful 投入與實踐生命的理想, 使我的生活有了意義與方向。	**5.19**	**1.01**	**-0.26(0.09)**	**-0.36(0.17)**	**0.778**
5	I take responsibility for the decisions I make 我會對自己做的決定, 擔負起責任。	**5.64**	**0.93**	**-0.51(0.09)**	**-0.26(0.17)**	**0.748**
6	I need to accept direct responsibility for my current situation 我認為, 我需對自己的現狀負直接的責任。	**5.62**	**0.93**	**-0.59(0.09)**	**0.22(0.17)**	**0.709**
7	I believe my attitude can change my destiny 我認為, 我的態度可以改變我的命運。	**5.49**	**1.02**	**-0.56(0.09)**	**0.02(0.17)**	**0.763**
8	I believe that a good life depends on my own effort 我相信擁有美好人生的關鍵, 在於自己的努力。	**5.46**	**1.04**	**-0.75(0.08)**	**0.87(0.17)**	**0.748**
9	I am willing to spend time with people who need comforting 我願意花時間陪伴需要安慰的人。	**5.45**	**0.93**	**-0.58(0.09)**	**0.42(0.17)**	**0.759**
10	I find meaning in my life by caring for and helping others 藉由關懷與助人, 讓我找到自己生命的意義與價值。	**5.40**	**0.97**	**-0.59(0.09)**	**0.35(0.17)**	**0.817**
11	I can selflessly care for and love those in need 對於需要幫助的人, 我能無私地付出自己的 關懷與愛。	**5.29**	**0.98**	**-0.39(0.09)**	**-0.01(0.17)**	**0.753**
12	I acquire affirmation and joy from giving 我由付出中獲得自我的肯定與喜悅。	**5.40**	**0.97**	**-0.43(0.09)**	**-0.12(0.17)**	**0.796**
13	I often think that being alive is something worthy of happiness 我常覺得能活著就是一件值得快樂的事情。	**5.44**	**1.05**	**-0.49(0.09)**	**0.01(0.17)**	**0.809**
14	I know why I live and for whom I am living for 我知道我為誰而活, 為何而活。	**5.31**	**1.10**	**-0.64(0.09)**	**0.49(0.17)**	**0.805**
15	I love my life 我熱愛我的生命。	**5.43**	**1.05**	**-0.48(0.09)**	**-0.07(0.17)**	**0.848**
16	I know that I am unique and that my existence is of great significance to some people 我知道我是獨一無二的, 我的存在對某些人是意義重大的。	**5.40**	**1.09**	**-0.53(0.09)**	**0.17(0.17)**	**0.824**
17	Because death is inevitable, I cherish every day 因為會死亡, 所以我珍惜每一天。	**5.43**	**1.05**	**-0.49(0.09)**	**0.07(0.17)**	**0.841**
18	I can’t decide when and how to die, but I can decide how to live every day 我雖然無法決定死亡, 但我可以決定如何過 每一天。	**5.51**	**0.99**	**-0.37(0.09)**	**-0.54(0.17)**	**0.845**
19	I hope to be able to say that I have lived a good life with no regrets at my death bed 我希望在生命的最後一刻能告訴自己, 我的一生活得很滿意而且沒有遺憾。	**5.48**	**1.08**	**-0.56(0.09)**	**0.01(0.17)**	**0.811**
20	I will live actively and happily later in my life 縱使到了人生的晚年, 我依舊會積極、快樂 的過生活。	**5.45**	**1.00**	**-0.46(0.09)**	**-0.27(0.17)**	**0.867**
21	I look forward to being tested by some setbacks in life 我希望在生命的旅程中可以經歷一些挫折與考驗。	**5.10**	**1.12**	**-0.58(0.09)**	**0.40(0.17)**	**0.723**
22	I that believe I can overcome obstacles in life 我相信我能克服生命的困境。	**5.31**	**0.97**	**-0.39(0.09)**	**-0.05(0.17)**	**0.831**
23	I view setbacks as life challenges and opportunities for growth 我把遭遇困境當成是生命的挑戰與成長的機會。	**5.29**	**1.04**	**-0.62(0.09)**	**0.71(0.17)**	**0.828**
24	I have gained valuable experiences from setbacks 在挫折裡, 我獲得很多寶貴的人生經驗。	**5.43**	**1.03**	**-0.54(0.09)**	**0.37(0.17)**	**0.823**

^**a**^The correlation coefficient between the item and the total of all items (with exception of the item)

### Factor analysis and construct validity

**Fig. 1:**
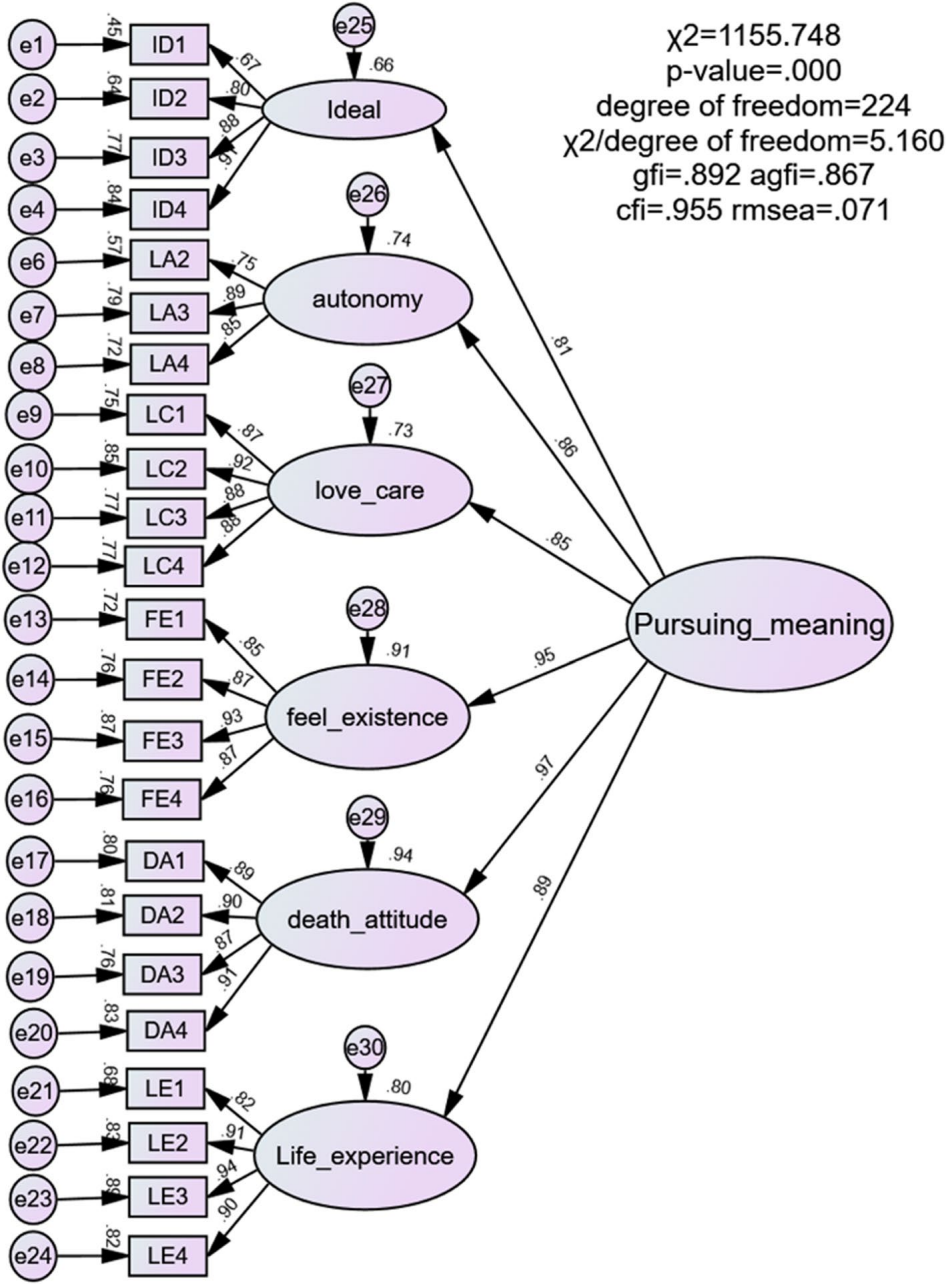
Structure of SFLAI-R: modified model of second-order confirmation factor analysis

We conducted CFA to examine the suitability of the hypothetical six-factor structure of the SF-LAI. Table 3 presents the goodness of fit values of the testing models. Overall, the six-factor model of the SF-LAI was confirmed. According to modification indices, we rechecked item 5 and agreed that it may be a replicate. Thus, item 5 was eliminated to improve model fit. Following the first-order CFA, the high correlation between feel existence and death acceptance implied that a single factor would be associated with the items of feel existence and death acceptance constructs. Therefore, we attempted to combine these two factors into one factor. However, the model fit indices of the five-factor model yielded no improvement in accuracy. Thus, a second-order factor analysis was performed to examine whether all factors were contributed by the common factor of the SF-LAI. Although the second-order fit indices were slightly lower than the first-order fit indices, they were within an acceptable range. The value of *T* = 0.90 implied the second-order model fit the data as well as the first-order model did. Because the second-order factor structure reduced correlations between the measurement errors of the first-order model and was more parsimonious and closely aligned with our present theoretical concepts of life attitude, we retained the second-order model for the remaining analyses (Fig. 1).

**Table 3:** Model fit indices for the CFA of SFLAI-R

Model	χ.^2^	*df*	χ^2^/ *df*^*a*^	CFI^b^	TLI^c^	RMSEA^d^ (90% CI)	SRMR^e^
Hypothesized six-factor	1242.110	237	5.241	0.953	0.946	0.075 (0.067 ~ 0.075)	0.032
five-factor	1405.205	242	5.807	0.946	0.938	0.076 (0.072 ~ 0.081)	0.033
Hypothesized six-factor (modified)	1040.042	215	4.837	0.960	0.953	0.068 (0.064 ~ 0.072)	0.032
Second-order Hypothesized six-factor (modified)	1155.748	224	5.160	0.955	0.952	0.071 (0.067 ~ 0.075)	0.038

*Abbreviations df* Degrees of freedom, *CFI* Comparative fit index, *TLI* Tucker–Lewis index, *CI* confidence interval, *RMSEA* Root mean square error of approximation, *SRMR* Standardized root mean square residual

^a^Criterion for acceptance is < 5, as recommended

^b^CFI. Values > 0.90 are adequate

^c^TLI. Values of > 0.80 are acceptable

^d^RMSEA. A value of < 0.08 indicates good fit

^e^SRMR. A value of < 0.08 indicates good fit

For the final model (23 items, second-order), Table 4 illustrates the indices of convergent validity. The factor loading of all items and the values of all 6 factor AVE were ≥ 0.50 and less than those of the CR represented an acceptable convergent validity. Table 5 shows that the CI (± two standard errors) for the correlation estimates between the pair factors did not include 1.0 and the difference between the chi-square values (with 1 degree of freedom) for the fixed and free solutions for the six pairs, were all significant, thus indicated satisfactory discriminant validity.

**Table 4:** Convergent validity for the final revised SFLAI

Factor	Item	Factor loading	Standard Residual	R^2^	AVE (> 0.5)
**Ideal**	ID1	0.667	0.555	0.445	0.674
	ID2	0.802	0.357	0.643	
	ID3	0.878	0.229	0.771	
	ID4	0.915	0.163	0.837	
**Autonomy**	LA2	0.762	0.419	0.581	0.693
	LA3	0.881	0.224	0.776	
	LA4	0.850	0.278	0.723	
**Love_care**	LC1	0.867	0.248	0.752	0.790
	LC2	0.922	0.150	0.850	
	LC3	0.877	0.231	0.769	
	LC4	0.879	0.227	0.773	
**Feel_existence**	FE1	0.844	0.288	0.712	0.777
	FE2	0.869	0.245	0.755	
	FE3	0.936	0.124	0.876	
	FE4	0.875	0.234	0.766	
**Death_attitude**	DA1	0.898	0.194	0.806	0.761
	DA2	0.898	0.194	0.806	
	DA3	0.869	0.245	0.755	
	DA4	0.822	0.324	0.676	
**Life_experience**	LE1	0.822	0.324	0.676	0.802
	LE2	0.908	0.176	0.824	
	LE3	0.943	0.111	0.889	
	LE4	0.904	0.183	0.817	

*R*^*2*^: Correlation coefficient, *AVE=* Average variance extracted

**Table 5:** Discriminant validity for the final model

Factor	ID	LA	LC	FE	DA	LE
ID	1					
LA	(122.313)^*^ 0.773^b**^ [.719, .817]^c^	1				
LC	(114.216)^*^ 0.702^**^ [.641,.751]	(123.680)^*^ 0.816^**^ [.760, .862]	1			
FE	(75.008)^*^ 0.760^**^ [.704, .805]	(101.700)^*^ 0.796^**^ [.746, .840]	(74.104)^*^ 0.797^**^ [.753, .841]	1		
DA	(60.344)^*^ 0.772^**^ [.727, .813]	(82.551)^*^ 0.815^**^ [.770, .858]	(59.068)^*^ 0.808^**^ [.763, .847]	(17.043)^*^ 0.943^**^ [.918, .962]	1	
LE	(74.311)^*^ 0.719^**^ [.659, .768]	(97.615)^*^ 0.762^**^ [.708, .812]	(65.711)^*^ 0.786^**^ [.745, .825]	(34.737)^*^ 0.841^**^ [.794, .879]	(21.538)^*^ 0.866^**^ [.825, .899]	1

^a^Chi-square differences provided in parentheses

^**b**^Paired correlations, ϕ

^c^Confidence interval of (ϕ ± 2 σ_e_) provided in parentheses

^*^p < .001

^**^p = .001

### Reliability assessment

Reliability coefficients for the SFLAI-R are presented in Table 6. Because our final model is a second-order scale. In addition to report CA, ω_*t*_, hierarchical omega (ω_*h*_*)* was also illustrated. When applied to the systematic variance attributable to multiple common factors, ω_*t*_ and ω_*t*_s are reported for general and group factors, respectively. On the otherhand, ω_*h*_ and ω_hs_ coefficients are reported as indicators of the systematic variance explained by a single general or group factor, respectively. The ω_*h*_ coefficient of 0.94 for the SFLAI-R indicates that 94% of the variance of unit-weighted SFLAI-R scores can be attributed to individual differences on the general life attitude factor. A comparison of ω_*t*_ (variance due to general and group factors) and ω_*h*_ (variance due to general factor alone) coefficients reveals that almost all of the reliable variance in SFLAI-R scales can be attributed to the general factor (0.94 ÷ 0.98 = 0.96). Thus, the SFLAI-R can confidently be interpreted as a reliable estimate of life attitude [66].

**Table 6:** Reliability indices for the final revised SFLAI

Factor	α	ω_*t*_/ω_tS_^*^	ω_*h*_/ω_hs_^**^
Ideal	0.88	0.94	0.08
Autonomy	0.87	0.94	0.24
Love_care	0.92	0.89	0.28
Feel_existence	0.93	0.94	0.19
Death_attitude	0.94	0.88	0.21
Life_experience	0.94	0.94	0.07
Total revised SFLAI scale	**0.97**	**0.98**	**0.94**

*α* Cronbach alpha coefficient, *ω*_*t*_ McDonald total omega, *ω*_*h*_ McDonald hierarchical omega

^*^ω_*t*_ and ω_*tS*_ are the omega coefficients for general and group factors, respectively

^**^ω_*h*_ and ω_*h*s_ are the omega hierarchical coefficients for general and group factors, respectively

### Measurement invariance

The results of the measurement invariance across genders and professions are displayed in Table 7. Based on the results of the configural model, we can conclude that no significant differences exist in the conceptualized factors between gender and profession. The metric invariance indicates that male and female respondents and physicians, nurses, and other hospital staff answered the items in a similar manner. The scalar invariance implies that we can compare means of factors across groups meaningfully [63].

**Table 7:** Measurement invariance for the final second-order six-factor model with respect to gender and profession

	**χ2 (*df*)**	**Δχ2 (Δ*df*)**	**p**	**CFI**	**TLI**	**RMSEA [90% CI]**	**ΔCFI**	**ΔTLI**	**ΔRMSEA**
**Gender**									
Configural invariance	1743.029 (448)	-	-	0.939	0.931	0.059 (0.056 ~ 0.062)	-	-	-
Metric invariance of the first-order factors	1779.959 (465)	36.930 (17)	0.003	0.938	0.933	0.058 (0.055 ~ 0.061)	-0.001	0.002	-0.001
Scalar invariance of the first- order factors	1799.242 (482)	19.283 (17)	0.313	0.938	0.935	0.057 (0.054 ~ 0.060)	0.000	0.002	-0.001
Metric invariance of the first- and second-order factors	1812.265 (487)	13.023 (5)	0.023	0.938	0.935	0.057 (0.054 ~ 0.060)	0.000	0.000	0.000
Scalar invariance of the first- and second-order factors	1821.536 (492)	9.271 (5)	0.159	0.937	0.936	0.057 (0.054 ~ 0.060)	-0.001	0.001	0.000
**Profession**									
Configural invariance	2289.901 (672)	-	-	0.926	0.917	0.054 (0.051 ~ 0.056)	-	-	-
Metric invariance of the first-order factors	2375.329 (706)	85.427 (34)	0.000	0.924	0.918	0.053 (0.051 ~ 0.056)	-0.002	0.001	-0.001
Scalar invariance of the first- order factors	2441.695 (740)	66.366 (34)	0.000	0.922	0.920	0.053 (0.050 ~ 0.055)	-0.002	0.002	0.000
Metric invariance of the first- and second-order	2473.595 (750)	31.901 (10)	0.000	0.921	0.920	0.053 (0.050 ~ 0.055)	-0.001	0.000	0.000
Scalar invariance of the first- and second-order factors	2495.292 (760)	21.697 (10)	0.017	0.921	0.921	0.052 (0.050 ~ 0.055)	0.000	0.001	-0.001

*Abbreviations*: *χ*^*2*^ Chi square, *df* Degrees of freedom, *Δχ*.^*2*^ Difference between the chi square values, *Δdf* Difference between degrees of freedom, *CFI* Comparative adjustment index, *TLI* Tucker–Lewis index, *RMSEA* Mean square root of the approximation error, *ΔCFI* Difference between the CFI, *ΔTLI* Difference between the TLI, *ΔRMSEA* Difference between the RMSEA

## Discussion

Training socially responsive medical professionals is a broad aim of medical education. Professionalism, humanism, and compassion are essential traits that HCWs should possess to meet the needs of the patients, especially in our aging society. However, these features are difficult to describe and assess. Inui and Swick proposed that the roots of professionalism are to be found in basic human values [67, 68], where the concept of “value” defined in terms of an individual’s attitude to their life and the world around them [69]. A positive attitude is necessary to motivation, engagement, a respect for human individuality, and a commitment to the betterment of humanity as a whole [70]. In this study, we revised and examined the factorial validity, construct validity, and internal reliability of the revised SF-LAI (SFLAI-R) to measure the attitude toward life among hospital staff of a teaching medical center in Taiwan. The psychometric results indicated that the SFLAI-R is a reliable and valid instrument to evaluate the life attitude of HCWs.

Our study reports that the six dimensions of the SF-LAI appropriately represent the underlying factor structure of HCWs’ life attitudes. These results are consistent with the original theoretical model proposed by Hsieh and Pan, which was applied on undergraduate students [39]. However, ours is a second-order scale, which means there presented a common factor, pursuing meaning. Comparing with western culture, the number of factors of our SF-LAI-R is comparable within 3 to 6. However, the contents are somewhat different. Of our six factors, the factor “ love and care” does not appear in the western scale, neither related words nor related meaning. In western culture, they don’t consider “ love and care”as part of life attitude. It might be due to the difference of individualism and collectivism. Besides, “death attitude”was sometimes omitted in the western life attitude scale. Because our scale is for HCW, we think these two are important elements of life attitude, we retain both of them.

The instrument also measures the construct among men and women (i.e., with respect to gender) and among physicians, nurses, and other hospital staff (i.e., with respect to profession) in a similar manner. The absence of significant deterioration in the model when factor loads and intercepts are restricted suggests that first, each item contributes to the latent construct to a similar degree across groups and second, that for the same score on the latent variable, people in the various groups do not have inconsistent means for the observed variables. That means that the differences observed in the results of the items are explained by differences in latent variables but not from the differences in the interpretation or meaning of the items. The results make it possible to compare the means between men and women and between physicians, nurses, and other hospital staff at the level of latent variables [71].

This study has several strengths. First, to our best knowledge, this is the first time the SFLAI-R has been distributed to HCWs in a teaching medical center. HCWs communicate the most with hospital patients who are often faced with choices that implicate their values and beliefs. Therefore, HCWs’ attitudes toward life are essential in their support of patients. The ability to reliably measure HCWs’ attitudes toward life is necessary to provide a framework for understanding and conceiving strategies to effectively inspire compassion at the bedside in the clinic and throughout the hospital.

Second, this study provides robust evidence that the SFLAI-R as a measurement of HCWs’ attitudes toward life is generalizable across genders and professions and has excellent internal consistency. Furthermore, the participant of this study was anonymous. It may improve the honesty of the respondents by reducing the social desirability especially in case of highly sensitive questions [72].

Finally, this study can allow researchers and educators to measure, compare, and identify the specific factors that would influence life attitude and help them develop empirical interventions to promote a positive attitude toward life.

This study also has several limitations. First, the response rate was less than 30%. We do not know how this instrument might function with non-responders. However, non-respondent might also be an expression of life attitude. It was proposed that survey respondents were more likely to be socially engaged [73]. In our study, male, physician and new staff showed significantly lower response rate. Although, there are many possibilities, indifference and negative life attitude might be one of them. The response rate may be used as an indicator of effectiveness of future humanistic education. Second, the cross-sectional and anonymous study design limited an examination of test–retest reliability. This is a clear drawback of the anonymous nature of online survey. Methods of tracking participants without compromising anonymity would help resolve this issue. Third, the study was conducted in a single teaching medical center in Taiwan. Future research may adopt a cross-hospital or cross-cultural research design. Finally, we did not display the empirical evidences to show the concurrent validity of the scale and empirical validity to support the real utility of this study. Reviewing of our final second-order scale, the common factor comes out to be persuing meaning, may be the Steger’s Meaning in Life Questionnaire (MLQ) could be used to evalaute the concurrent validity [74] in the future. Because life attitude is a complex, multilevel concept; external data for use as a standard for comparison is lacking. Future studies might compare the outcomes of the SFLAI-R with patient satisfaction, quality of care, or hospital accreditation.

## Conclusions

In conclusion, with the evolution in health care demands, HCWs’ should appreciate the intangible concept of human value. To create effective educational strategies and curricula, we require a valid instrument to explore the attitude toward life among HCWs. The SFLAI-R exhibits excellent construct validity and internal consistency to measure life attitude and could therefore be used to measure the differences and teaching effectiveness among HCWs exposed to an experimental curriculum in humanistic teaching practices in the future.

## Data Availability

The data used and/or analysed during the current study are available from the corresponding author on reasonable request.

## Abbreviations

SF-LAI: Short-Form Life Attitude Inventory
CFA: Confirmatory factor analysis
CFI: Comparative fit index
TFI: Tucker–Lewis index
SRMR: Standardized root mean square residual
RMSEA: Root mean square of error of approximation
WPC: Whole person care
HCWs: Healthcare workers
LAP: Life Attitude Profile
MiL: Meaning in life
LAP-R: Life Attitude Profile-Revised
LAI: Life Attitude Inventory
AVE: Average variance extracted
CR: Composite reliability
CI: Confidence interval
SFLAI-R: Revised SF-LAI
